# Validation of the *SHOX2*/*PTGER4* DNA Methylation Marker Panel for Plasma-Based Discrimination between Patients with Malignant and Nonmalignant Lung Disease

**DOI:** 10.1016/j.jtho.2016.08.123

**Published:** 2017-01

**Authors:** Gunter Weiss, Anne Schlegel, Denise Kottwitz, Thomas König, Reimo Tetzner

**Affiliations:** Epigenomics AG, Berlin, Germany

**Keywords:** *SHOX2*, *PTGER4*, DNA methylation, Lung cancer early detection, Circulating tumor DNA, Liquid biopsy

## Abstract

**Introduction:**

Low-dose computed tomography (LDCT) is used for screening for lung cancer (LC) in high-risk patients in the United States. The definition of high risk and the impact of frequent false-positive results of low-dose computed tomography remains a challenge. DNA methylation biomarkers are valuable noninvasive diagnostic tools for cancer detection. This study reports on the evaluation of methylation markers in plasma DNA for LC detection and discrimination of malignant from nonmalignant lung disease.

**Methods:**

Circulating DNA was extracted from 3.5-mL plasma samples, treated with bisulfite using a commercially available kit, purified, and assayed by real-time polymerase chain reaction for assessment of DNA methylation of short stature homeobox 2 gene (*SHOX2*), prostaglandin E receptor 4 gene (*PTGER4*), and forkhead box L2 gene (*FOXL2*). In three independent case-control studies these assays were evaluated and optimized. The resultant assay, a triplex polymerase chain reaction combining *SHOX2*, *PTGER4*, and the reference gene actin, beta gene (*ACTB*), was validated using plasma from patients with and without malignant disease.

**Results:**

A panel of *SHOX2* and *PTGER4* provided promising results in three independent case-control studies examining a total of 330 plasma specimens (area under the receiver operating characteristic curve = 91%–98%). A validation study with 172 patient samples demonstrated significant discriminatory performance in distinguishing patients with LC from subjects without malignancy (area under the curve = 0.88). At a fixed specificity of 90%, sensitivity for LC was 67%; at a fixed sensitivity of 90%, specificity was 73%.

**Conclusions:**

Measurement of *SHOX2* and *PTGER4* methylation in plasma DNA allowed detection of LC and differentiation of nonmalignant diseases. Development of a diagnostic test based on this panel may provide clinical utility in combination with current imaging techniques to improve LC risk stratification.

## Introduction

Lung cancer (LC) remains one of the world’s most common and deadliest forms of cancer.^1^ Screening with low-dose computed tomography (LDCT) has been shown to reduce LC mortality.^2^ Consequently, most U.S. medical societies recommend screening with LDCT for certain high-risk groups defined mainly by age and a history of intense smoking.^3^ When the original definition for a positive screen from the United States–based National Lung Screening Trial is used, LDCT performance suffers from a substantial number of positive calls (27%), of which 96% have been determined to be false positives.^4^ As a consequence, the NELSON trial used a substantially different definition of a positive screening result, which led to a 10-fold decrease in the positive rate (2.7%), reducing the proportion of false positives to 60% at the expense of some reduction in sensitivity for LC detection.^5^, ^6^ The need for better definition of the screening-eligible population led to risk assessment models developed from large trials.^7^, ^8^ Similarly, algorithms for management of so-called intermediate nodules have been published.^9^, ^10^, ^11^ Because of the imperfections of each of these methods, there is an ongoing quest for sensitive and reliable biomarkers with the potential to complement current cancer risk assessments.^12^

The epigenetic events occurring at early stages of carcinogenesis are a source of biomarkers.^13^ Aberrant DNA methylation has been extensively described as a means to aid in the detection of cancer, specifically, in specimens that are amenable for minimal invasive sampling.^14^ Only recently, DNA methylation–based biomarkers have been successfully incorporated into commercially available in vitro diagnostic (IVD) devices approved by U.S. Food and Drug Administration for use in cancer screening.^15^, ^16^ For colorectal cancer screening in stool, a multitarget stool DNA test combined the information of a fecal immunochemical assay for human hemoglobin, mutations in the *KRAS* gene, and the DNA methylation markers bone morphogenic protein 3 gene (*BMP3*) and NDRG family member 4 gene (*NDRG4*).^17^, ^18^ Screening for colorectal cancer in a simple blood draw was made possible with real-time polymerase chain reaction (PCR) assessment of methylated Septin9 DNA derived from plasma.^19^ Both of these methods have been validated in extensive prospective screening trials.^20^, ^21^, ^22^

In LC methylated short stature homeobox 2 gene (*SHOX2*) DNA has been described as a valuable biomarker in several research studies. Elevated *SHOX2* methylation was associated with detection of LC in bronchial aspirates, pleural effusions, and blood plasma.^23^, ^24^, ^25^ Consequently, an IVD intended as a diagnostic adjunct to existing clinical and pathological parameters was developed and validated on bronchial aspirate specimens. An additional clinical application of the biomarker *SHOX2* was the determination of LC stage via assessment of *SHOX2* positivity of lymph nodes.^26^ Also, the potential of *SHOX2* DNA methylation for monitoring the response to chemotherapy has been published recently.^27^

From the perspectives of the patient and the clinician, the use of simple blood draw as a primary sample is most appealing independent of the clinical application potential. Such a liquid biopsy is easily performed during routine clinical, whereas tissue biopsies are invasive and potentially erroneous for difficult-to-reach areas of the lung. Therefore, we have investigated further the potential of *SHOX2* and two additional methylation markers, forkhead box L2 gene (*FOXL2*) and prostaglandin E receptor 4 gene (*PTGER4*), for use as a noninvasive, blood plasma–based diagnostic tool to differentiate patients with LC from healthy subjects and patients with nonmalignant disease of the lung. Here we report on three independent case-control studies used to identify a minimal set of biomarkers with high sensitivity for LC and develop in parallel an assay format suited for an IVD product. Finally, we report on a validation study that includes patients with LC as well as patients with a variety of nonmalignant lung diseases.

## Materials and Methods

### Subjects

The patient samples were either material collected over the course of several months by commercial partners (ProteoGenex Inc., Sofia Bio LLC) or leftover material from previously conducted clinical trials in colon cancer.^21^, ^22^ More specifically, patients with lung disease (LC and benign disease cases) were collected under the same protocol at clinical sites in Bulgaria by Sofia Bio, LLC, or in Russia and the United States by ProteoGenex, Inc. The included healthy controls were collected at clinical sites in the United States. Study participants provided informed consent and all involved institutions adhered to the local ethical guidelines.

For three sequential training studies samples were randomly chosen from a collection of a total of 118 LC cases and 212 healthy control subjects. These LC cases covered all major histological types and a broad range of stages (Table 1). The first small pilot study (study 1) comprised 10 LC cases and 20 control subjects. The remaining 300 samples were randomized into 23 processing batches. Study 2 used the first 12 batches, which comprised a total of 151 samples (59 LC cases). Study 3 used the remaining 11 batches, which contained 149 samples (49 LC cases). The validation study was conducted with 72 healthy controls, 50 patients with nonmalignant lung disease, and 50 patients with LC. These patients with LC were a random subset from patients in studies 2 and 3 with a second plasma aliquot available that was restricted to cases from the provider (Sofia Bio, LLC), which also provided the 50 nonmalignant lung disease samples.

**Figure 1:**
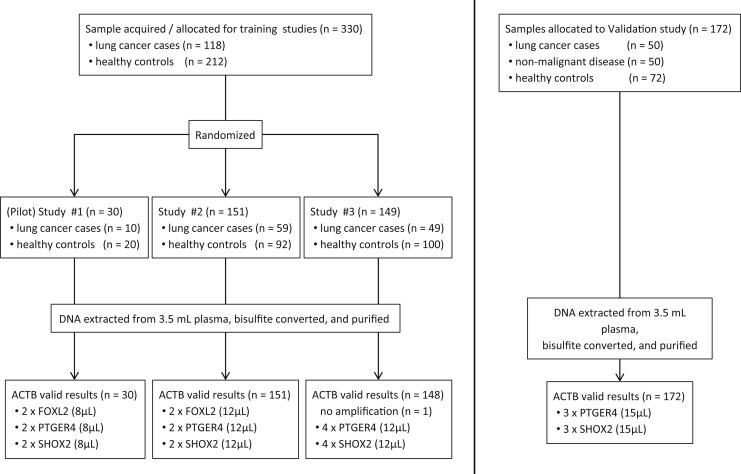
Sample disposition, study setup, and polymerase chain reaction (PCR) assay formats. Boxes in the bottom line indicate number of valid results, number of PCR replicates, and bisDNA input volume (in parenthesis) per PCR assay. For more details, see Materials and Methods section. *ACTB*, actin, beta gene; *FOXL2*, forkhead box L2 gene; *PTGER4*, prostaglandin E receptor 4 gene; *SHOX2*, short stature homeobox 2 gene.

Diagnosis of lung disease (malignant or nonmalignant) was provided by the participating institutions. The nonmalignant diseases were mainly asthma, chronic obstructive pulmonary disease, and pneumonia (Table 2). Figure 1 displays details of the sample disposition. Diagnostic information for cancer cases included information on histological type and disease stage. Control subjects were self-declared to be healthy as there was no specific assessment for lung disease as part of their study inclusion. Common demographic information was limited to age and sex. Smoking history was available for patients with LC and most of the patients with nonmalignant lung disease but for only 36 of the healthy controls.

### Blood Sampling, Sample Preparation, and DNA Methylation Analysis

At all institutions, blood sampling, and plasma preparation were performed according to the same protocol by trained staff.^28^ Specifically, blood was drawn in 10-mL BD Vacutainer ethylenediaminetetraacetic acid tubes (BD Biosciences, San Jose, CA). Plasma was prepared by a double-spin procedure within 4 hours after blood draw. Plasma was stored frozen at –80°C and kept frozen during shipment on dry ice to the testing laboratory.

All plasma samples had a bar code as their only identifier. Thus, the clinical group (LC or control) of the sample was masked to the laboratory personnel conducting the experiments.

At the testing site plasma samples were thawed and processed in batches together with positive and negative workflow controls using the Epi proColon Plasma Quick Kit according to the respective instructions for use.^28^ Briefly, circulating DNA was extracted from 3.5 mL of plasma by utilizing magnetic particles. Thereafter, the DNA was converted in a bisulfite reaction. After purification, bisulfite-converted DNA (bisDNA) was eluted in 60 μL and ready for use in real-time PCR.

The sequence of three training studies was conducted to evaluate and develop suitable combinations of methylation markers and respective real-time PCR assays. All target gene PCR assays used methylation-unspecific primer oligonucleotides (length 17–25 base pairs) and a blocker oligonucleotide (length 26–35 base pairs) to suppress the amplification of unmethylated target sequences.^29^ Hydrolysis probes specific for the methylated target sequence were designed for PCR detection on fluorescence channels FAM, Texas Red, and VIC. The PCR conditions (volumes, cycling program, and threshold settings) were essentially those detailed in the instructions for use of Epi proColon.^28^

For study 1, real-time PCR assays were designed for three methylation markers, *FOXL2*, *PTGER4*, and *SHOX2*. Each PCR assay was run in duplicate with an 8-μL bisDNA input volume. For study 2, two duplex assays were designed, the first comprising methylation markers *FOXL2* and *PTGER4* and the second comprising marker *SHOX2* and *ACTB* as an internal reference assay. Both assays were run in PCR duplicates with 12 μL of bisDNA input. For study 3, the marker *FOXL2* was dropped and a triplex assay for *PTGER4*/*SHOX2*/*ACTB* was designed. The triplex PCR assay was then run in quadruplicate with 12 μL of bisDNA input. Finally, the validation study used an optimized version of the triplex assay with 15 μL of bisDNA input run in triplicate. Figure 1 displays details of the study setup. The total reaction volume for all PCR assay studies was 30 μL. All assays were run on the Applied Biosystems 7500 FAST Real-Time PCR System (Applied Biosystems, Foster City, CA).

### Data Analysis

Real-time PCR data were analyzed using the Sequence Detection Software v1.4 21 CFR Part 11 Module (Thermo Fisher Scientific, Waltham, MA) with appropriate settings for assay thresholds and baseline window resulting in a cycle threshold (Ct) value per assay for each PCR well.

For each sample the minimal Ct value (minCt) aggregated over PCR replicates per PCR assay represented the respective measurement. During studies 1 through 3 simple classifiers were built by combining the assay results (minCt) through logistic regression. In the validation study, analysis was conducted by applying the trained model to the validation data.

Receiver operating characteristic (ROC) and the area under the ROC curve (AUC) were analyzed using the R environment (Version 3.1.2) (R Foundation for Statistical Computing, Vienna, Austria).^30^

## Results

**Figure 2:**
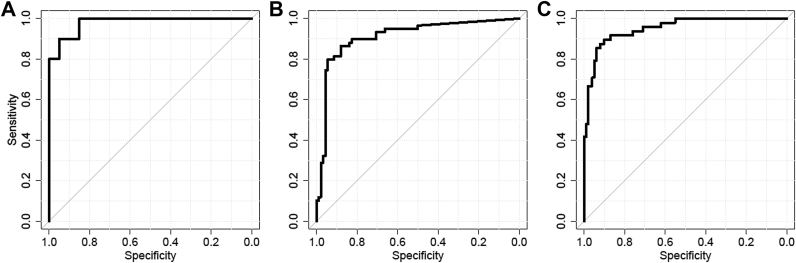
Receiver operating characteristic and area under the curve (AUC) analysis of pilot study 1 (AUC = 0.98) (*A*), study 2 (AUC = 0.91) (*B*), and study 3 (AUC = 0.95) (*C*).

Pilot study 1 was conducted with a set of three research PCR assays. Because this pilot study provided excellent discrimination of the LC group from the controls (AUC = 0.98 [Fig. 2*A*]), the assays were developed further into duplex PCR assays to allow for more efficient use of the bisDNA in PCR in a follow-up study. This assay format was used in study 2, which was conducted to evaluate the three-marker panel for its ability to differentiate patients with LC from healthy subjects. The 59 LC cases included there represented all major histological types of LC and a broad distribution of stages (IA to IV). On the basis of a logistic regression model using the minCt aggregation of the data, the marker panel demonstrated significant discriminatory power (AUC = 0.91 [Fig. 2*B*]). Detailed analysis led to the hypothesis that the marker FOXL2 could be dropped from the panel without significant loss of performance. This hypothesis was tested in study 3, which contained 49 LC samples among the 149 samples tested. Here, a single LC sample did not amplify efficiently in PCR analysis and was excluded from data analysis. Before conduct of study 3 the PCR assay format was changed to a triplex assay containing *PTGER4*, *SHOX2*, and *ACTB* as the final configuration. Optimized use of the bisDNA volume was achieved through this development. The substantial size of study 3, as well as the observed discriminatory power of the two-marker panel (AUC = 0.95 [Fig. 2*C*]), provided a convincing argument for reduction of the marker panel's complexity. The case and control groups differed substantially with regard to their sex distribution but were similar with regard to age (Table 1). Comparison of a regression model including *PTGER4*, *SHOX2*, and sex as predictors with a model including sex only confirmed the substantial discriminatory power of the methylation panel (*p* < 0.0001, likelihood ratio test).

**Figure 3:**
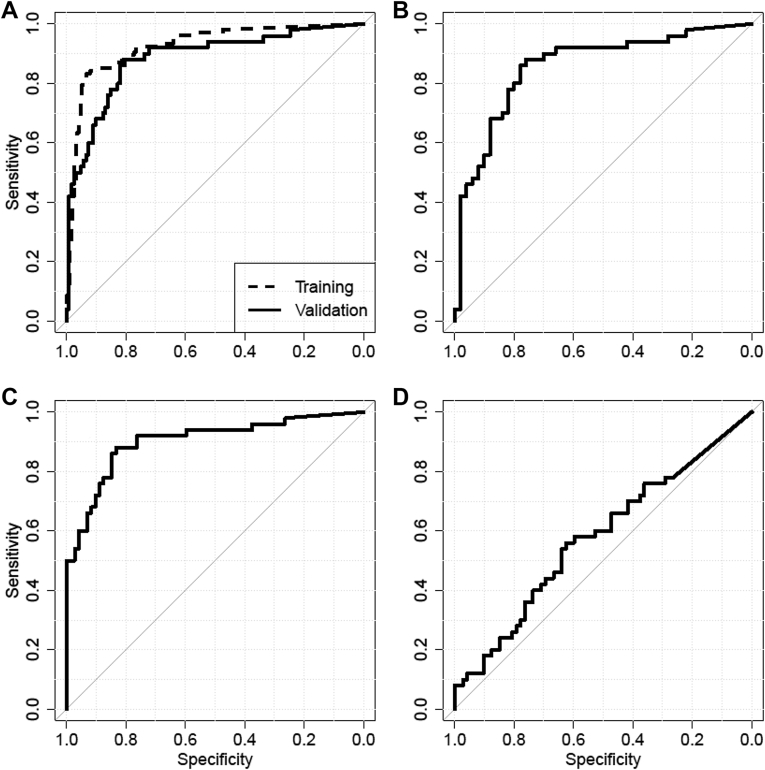
Receiver operating characteristic and area under the curve (AUC) analysis of validation study: (*A*) Lung cancer (LC) versus all controls for training (AUC = 0.93) and validation study (AUC = 0.88), (*B*) LC versus nonmalignant disease (AUC = 0.86), (*C*) LC versus healthy controls (AUC = 0.91), (*D*) nonmalignant disease versus healthy controls (AUC = 0.58).

Thereafter, the data from study 2 and 3 were collapsed and used to train a classifier by fitting a logistic regression model based on minCt-values. The trained model was used to analyze the data from the validation set, which comprised 50 LC cases and 122 subjects with or without a nonmalignant lung disease. The case and control groups were similar with respect to their median age, and sex bias was substantially smaller than in the training set (Table 2). The results of the ROC analysis for the model are displayed in Figure 3*A* for both the training set and the validation set. Performance of the model in the validation set was close to the previous results (AUC = 0.88 versus AUC = 0.93). At a fixed specificity of 90%, sensitivity for LC was 67%; at a fixed sensitivity of 90%, specificity was 73%. A detailed analysis of the data revealed good performance for comparison of the LC group with 50 patients with nonmalignant disease (AUC = 0.86 [Fig. 3*B*]) as well as with the healthy controls (AUC = 0.91 [Fig. 3*C*]). The nonmalignant disease group and the healthy controls were not different on the basis of their classifier values (AUC = 0.58, *p* = 0.15) (Fig. 3*D*). A final comparison was conducted in 36 healthy subjects with documented smoking history. The classifier did not distinguish the group of 12 smokers from the remaining 24 individuals (AUC = 0.56).

## Discussion

This study demonstrates that assessment of DNA methylation markers in blood plasma provides a very reliable diagnostic method. Of 502 plasma specimens processed in total over the course of four studies, 501 (99.8%) provided a valid result. Only a single instance of an invalid sample occurred. The two-marker panel demonstrated high discriminatory power to differentiate patients with LC from healthy subjects in training studies (AUC = 0.93). This panel was validated in a study incorporating patients with serious nonmalignant lung diseases such as chronic obstructive pulmonary disease into the control group. At the same time, improvements in the PCR assay format were implemented. The reliability of the approach is demonstrated by the results of the validation study (AUC = 0.88). There was no substantial performance loss observed despite the fact that more than 40% of cases in the control group were nonmalignant disease cases.

**Figure 4:**
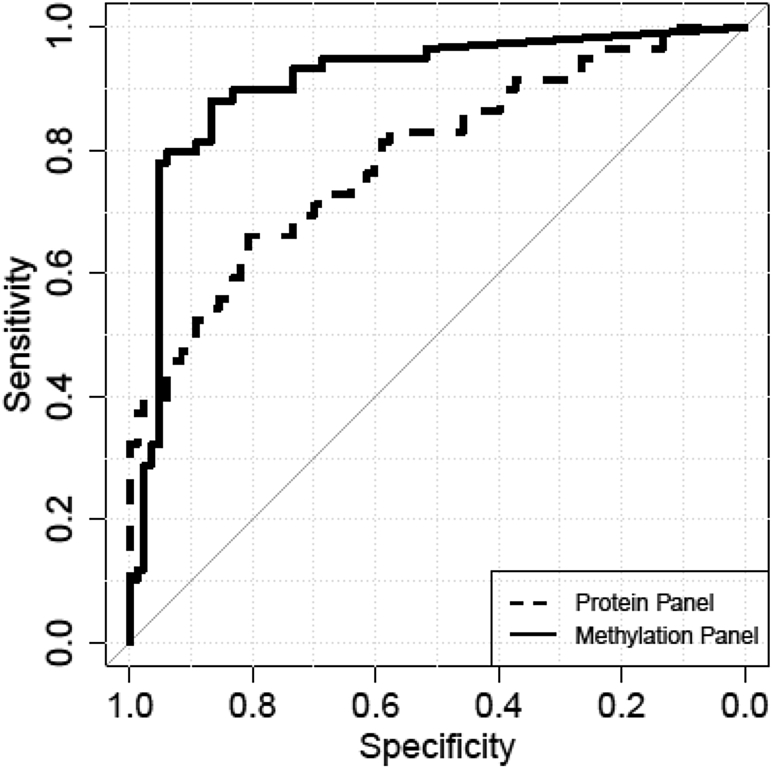
Comparison of protein (area under the curve [AUC] = 0.79) and methylation (AUC = 0.91) marker panel. The difference in the AUCs was statistically significant (*p* value = 0.004).

However, generalizability of the results of the validation study may be limited as the samples from diseased patients were collected by a single provider. In an attempt to mitigate the risk of reporting a singular observation, leftover specimens of study 2 were assessed for concentration of four proteins commonly reported as LC biomarkers (carcinoembryonic antigen, cytokeratin 19 fragment, cancer antigen 125, and carbohydrate antigen 19-9)^31^ by a commercial service provider. The protein marker panel was used to benchmark the result of the methylation biomarkers. Figure 4 displays the performance of this protein panel together with the two-marker methylation panel (*SHOX2*/*PTGER4*) in a side-by-side comparison. For this sample set, methylation information (AUC = 0.91) outperforms protein information (AUC = 0.79) with a statistically significant difference (*p* = 0.004).

A number of different types of plasma biomarkers have recently been explored as aids in the diagnostic work-up of patients with LC.^32^ Two laboratory-developed tests are currently available in the United States. One 11-protein classifier for management of lung nodules (Xpressys [Integrated Diagnostics, Inc., Seattle, WA]) demonstrated consistently diagnostic information of approximately 10% (Youden's index^33^ = Sensitivity + Specificity – 1 ≈ 10%).^34^, ^35^, ^36^ The performance results for a panel of autoantibodies for screening of high-risk patients, EarlyCDT-Lung (Oncoimmune, Ltd., Nottingham, United Kingdom), have been recently confirmed in a routine clinical setting (Youden's index ≈ 30%).^37^, ^38^ on different sets of microRNAs (miRNAs), promising results with a Youden's index of approximately 65% have been reported for both a 34-miRNA classifier in serum^39^ and a 24-miRNA signature classifier in plasma.^40^

The performance of the *SHOX2*/*PTGER4* marker panel as observed in the validation set (Youden's index ≈ 60%) appears to be competitive with results of other published methods. Used as a reflex test for LDCT screen–positive patients with a decision rule with excellent sensitivity, the test has reasonably high specificity that would lead to a substantial reduction of the false-positive rate of LDCT. Adopted in contrast to very high specificity, the test may even be useful as a minimally invasive screening test directing patients to screening with LDCT. However, more clinical data are needed to make a fair assessment of the strengths and weaknesses of a DNA methylation–based approach.

In conclusion, the result of this research validates the potential of the *PTGER4*/*SHOX2* methylation marker panel as a minimally invasive diagnostic method to discriminate between patients with and without malignant lung disease. Used as a complementary tool to current screening methods it may prove advantageous for selection of LDCT screening eligible individuals. Patients at increased risk on the basis of life history, symptoms, or findings in LCDT may eventually benefit from the information provided by a confirmatory assessment based on *PTGER4*/*SHOX2* methylation results. However, these clinical uses need to be validated in future clinical trials.

## Figures and Tables

**Table 1:** Patient Characteristics for Studies 1 to 3

Patients	Median Age (Range), y	Sex (% male)	Clinical Categories			
Lung cancer (n = 117)	64 (38 – 80)	77	Histological Subtype (n = 117 )			
			Adeno	Squamous	Other	SCLC
			46	58	8	5
			Stage (n = 113^a^)			
			0 / I	II	III	IV
			26	21	42	24
Healthy patients (n = 212)	61 (50 – 83)	27				

Adeno, adenocarcinoma; Squamous, squamous cell carcinoma.

a Four subjects with unknown stage.

**Table 2:** Patient Characteristics for Validation Study

Patients	Median Age (Range), y	Sex (% male)	Clinical Categories			
Lung cancer (n = 117)	63 (43 – 80)	77	Histological Subtype (n = 50 )			
			Adeno	Squamous	Other	SCLC
			18	25	7	-
			Stage (n = 50)			
			0 / I	II	III	IV
			12	11	16	11
Benign disease (n = 50)	68 (50 – 83)	62	Disease Group (n = 50)			
			Asthma	COPD	Pneumonia	Other
			5	18	11	16
Healthy patients (n = 72)	60 (49 – 80)	50	Smoking Status (n = 36^a^)			
			Smoker		Nonsmoker	
			12		24	

Adeno, adenocarcinoma; Squamous, squamous cell carcinoma; COPD, chronic obstructive pulmonary disease.

a Thirty-six subjects with unknown smoking status.
